# Molecular mechanisms regulating immune responses in thromboangiitis obliterans: A comprehensive review

**DOI:** 10.22038/ijbms.2019.31119.7513

**Published:** 2019-03

**Authors:** Abbas Shapouri-Moghaddam, Mohammad-Hadi Saeed Modaghegh, Hamid reza Rahimi, Seyyed-Morteza Ehteshamfar, Jalil Tavakol Afshari

**Affiliations:** 1 Immunology Research Group, Bu-Ali Research Institute, Mashhad University of Medical Sciences, Mashhad, Iran; 2 Vascular and Endovascular Surgery Research Center, Alavi Hospital, Mashhad University of Medical Sciences, Iran; 3 Neurogenic Inflammation Research Center, Mashhad University of Medical Sciences, Mashhad, Iran; 4 Department of Modern Sciences and Technologies, Faculty of Medicine, Mashhad University of Medical Sciences, Mashhad, Iran; 5 Immunology Research Group, Faculty of Medicine, Mashhad University of Medical Sciences, Mashhad, Iran

**Keywords:** Angiogenesis, Immune system, Molecular biology, Signal pathways, Thromboangiitis obliterans

## Abstract

Thromboangiitis obliterans (TAO) is a thrombotic-occlusive as well as an inflammatory peripheral vascular disease with unknown etiology. Recent evidence has supported the immunopathogenesis of the disease, however, the factors contributing to the altered immune function and vascular tissue inflammation are still unclear. This review was intended to collate the more current knowledge on the regulatory molecules involved in TAO from an immunoreactive perspective. The homeostasis of the immune system as well as a variety of progenitor cell populations appear to be affected during TAO and these alterations are associated with intrinsic signaling defects that are directing to an improved understanding of the crosstalk between angiogenesis and the immune system, as well as the potential of new co-targeting strategies applying both immunotherapy and angiogenic therapy.

## Introduction

Thromboangiitis obliterans (TAO) is described as a non-atherosclerotic segmental inflammatory and occlusive vascular disease which mainly involves the small- and medium-sized arteries, veins, and nerves of the upper and lower extremities (1). Despite intriguing advances in the clinical understanding of TAO (2-5), there is still a lack of detailed reports on the molecular evidence underlying immune responses in the disease. Hitherto, four factors have been identified to affect the pathogenesis of Buerger’s disease: variant of atherosclerosis; immunologic arteritis; odontal bacterial thrombosis; hyperhomocysteinemia. Thus, Buerger’s disease is representative of immune-mediated arteritis based on the immunocytochemical evidence, characterized by the accumulation of immunoglobulins and complement factors in the vessel wall (6). Moreover, some have reported an elevation of cellular immunity to type I and type III collagens (7). The presence of antiendothelial antibodies has been determined, which is indicative of the immune response towards the endothelium (8). Such findings have drawn attentions towards the role of the immune system in the development of TAO. This review aimed at elucidating the molecular mechanisms implicated in the dysregulation of the immune response during the disease process.

**Figure 1:**
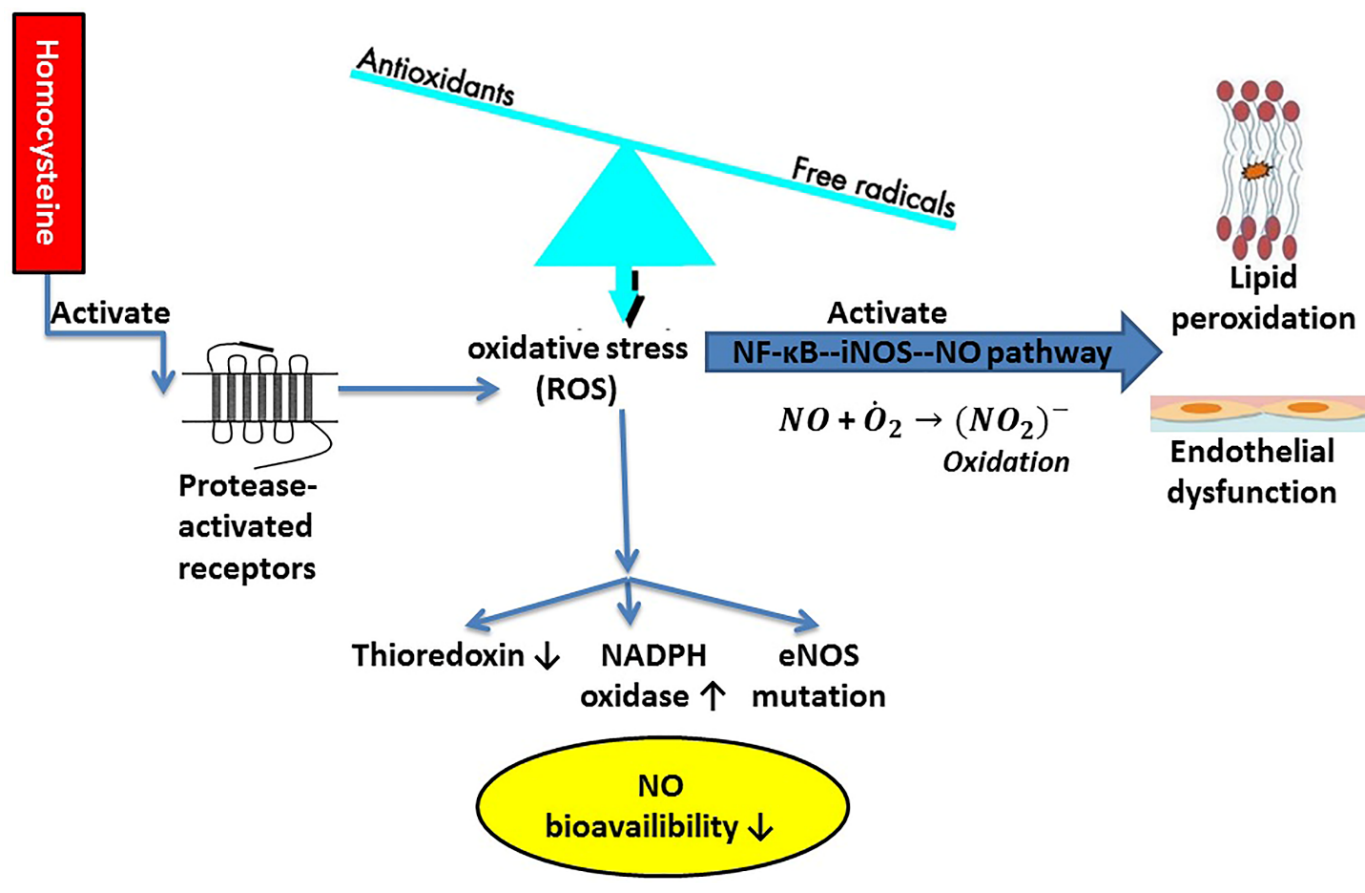
Oxidative stress through the stimulation of the endothelial NF-ĸB--iNOS--NO pathway and upregulation of homocysteine; eNOS: endothelial nitric oxide synthase, iNOS: inducible nitric oxide synthase, NADPH: nicotinamide adenine dinucleotide phosphate, NF-κB: nuclear factor kappa B, NO: nitric oxide, ROS: reactive oxygen species

**Figure 2:**
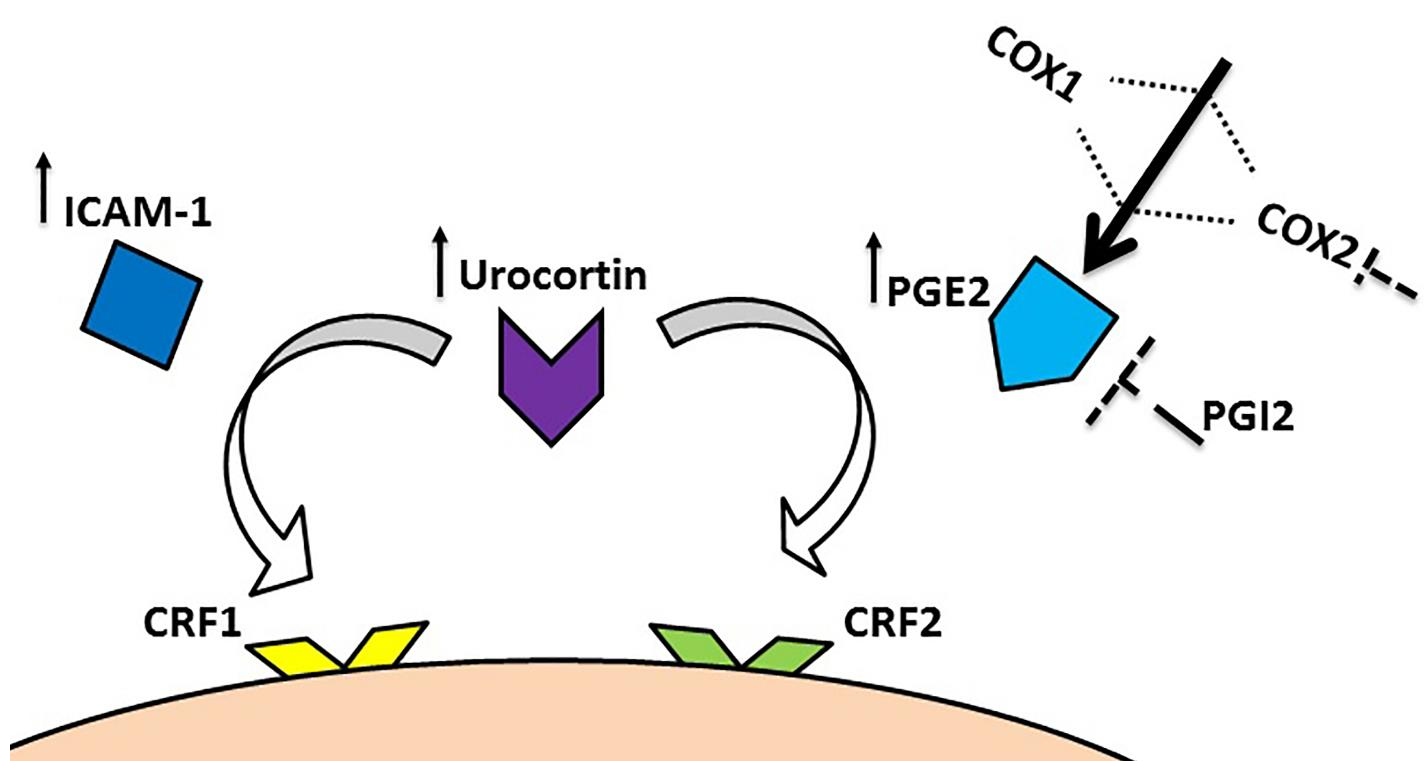
Schematic presentation of COX inflammatory pathway; COX: cyclooxygenase,CRF: corticotrophin-releasing factor, ICAM-1: intercellular adhesion molecule-1, PG: prostaglandin

**Oxidative stress**

It has been indicated that oxidative stress contributes to the development of TAO. Indeed, the oxidative stress level was found on the considerable rise in the TAO patients as opposed to the healthy smokers and non-smokers (9). Moreover, oxidative stress is more likely to come about via the stimulation of the endothelial nuclear factor-kappa B-inducible nitric oxide synthase-nitric oxide (NF-ĸB--iNOS-NO) pathway, leading to the additional release of nitric oxide (NO) that can react with the superoxide anion and induce the strong oxidation of nitrite anion, which, in turn, comes up with lipid peroxidation and direct endothelial dysfunction (Figure 1). The expression of adhesion molecules and inflammatory factors were elevated, as well (10). Indeed, oxidative materials present in cigarette are more likely to impair the function of the platelets and vascular endothelial cells, thus increasing the thrombotic events as well as allowing for the inflammatory reactions, known as the two leading events in TAO (11). Smoking can cause brief leukocytosis and a delayed elevation of neutrophil action (12). The generation of the oxygen-derived free radicals from the stimulated neutrophils may underlie TAO through changes in the endothelial morphology or function, thereby increasing the expression of adhesion molecules and giving rise to the risk of thrombosis as well as inflammation (13, 14). The assessment of pro-oxidant–antioxidant balance (PAB) shows the balance of oxidants and antioxidants at the same time in one experiment (15). A low PAB value indicated the higher concentration of antioxidants than oxidants and vice versa (15). In the study by Arslan *et al.* (9), it was found that the oxidative stress markers, including plasma protein carbonyls, serum ox-LDL, and plasma and erythrocyte malondialdehyde (MDA), substantially increased in the TAO patients in comparison with the healthy controls. In contrast, the antioxidant markers, namely NO, plasma paraoxonase levels, glutathione and glutathione peroxidase levels, superoxide dismutase, and catalase levels, in erythrocytes appeared in a significant decline in the TAO patients versus the control group, who included only healthy non-smokers. Therefore, these findings might be the result of cigarette smoking. Additionally, there has been some evidence of hyperhomocysteinemia in TAO (16, 17). Homocysteine is correlated with malnutrition to a great extent (18), carries a stimulatory impact on the protease-activated receptors, and then elevates the generation of reactive oxygen species (ROS) via the promotion of the nicotinamide adenine dinucleotide phosphate (NADPH) oxidase and yet the diminution of the thioredoxin expression that results in the reduction of the NO bioavailability in the cardiac microvascular endothelial cells (Figure 1) (19). This was emphasized by Arslan *et al.* (9), whose study exhibited considerably lower levels of NO in the TAO patients than the control group. In general, NO serves as a free radical scavenger, preventing the accumulation of platelets and adhesion of endothelial cells during the inflammatory events, and promoting a normal blood flow (11). The reduced levels of NO in TAO can be related to cigarette smoke (11), where the ROS exerts a lowering effect on the NO availability by its direct removal or disruption in its biosynthesis, and on the endothelial nitric oxide synthase (eNOS) activity (20). The report concerning a mutation of the T-786C eNOS gene in the TAO patients may pinpoint the influences of oxidative stress caused by cigarette smoke on the NO bioavailability (16). These events can justify damages to the endothelium-dependent vasodilation recorded in TAO, as well (21). Oxidative stress is a primary factor that, upon its activation, the function or morphology of endothelial cells can be impaired, which often results in thrombosis as well as inflammation. However, the genetics or epigenetics of the pathways involved in oxidative stress are unknown in TAO. Antioxidant therapy by known genetic variations in antioxidants, known epigenetic alterations, and probably certain biomarker profiles should be investigated for its effects on the reduction of the homocysteine level in TAO patients.

**T cell-related immune injury**

A body of literature has addressed TAO as an autoimmune disease (22). However, the current knowledge is limited and more evidence is required to identify all immune disorder processes responsible for TAO immunopathogenesis (23). The TAO patients have presented with marked serum levels of various immune complexes, such as anti-endothelial cell antibodies, anti-neutrophil antibodies (ANA), and anti-cardiolipin antibodies (ACA), in reference to the healthy subject (24). Similarly, Maslowski *et al.* (25) found out that ACA can play a key role in TAO. Nevertheless, the patients who had generalized periodontitis presented with remarkably higher titres of immunoglobulin G (IgG) or IgM ACA, which were on the notable rise in the smokers more than the non-smokers (26). Guo *et al.* (24) also confirmed the abnormality of ANA, ANA specificity antigens, and ACA in the TAO patients. Indeed, ANA as well as ACA may be respectively correlated with immunolesion, and thrombosis as well as angiitis caused by TAO. The underlying mechanisms ascribed to the role of ANA in the progression of immunolesion of blood vessels are as follow: the presence of ANA has a stimulative effect on neutrophils to generate ROS and lytic enzymes that further induce the lysis of endothelial cells along with neutrophils (27, 28); the ANA IgG can carry influences on many neutrophil functions, such as, the enhancement of a proinflammatory phenotype whereby neutrophils are able to increase the collateral damage in endothelial and other cells (29); lastly, ANA can cause the disturbance of the T-cell compartment and alteration in the T-cell subsets that are influential on the T-cell immunological function and accompanied by the direct damage to endothelial cells (30). When it comes to ACA, the pathogenesis of TAO arises from three main actions (31, 32): ACA can affect the monocytes and vascular endothelial cells in such a way that secrete tissue factor with a capability of triggering hypercoagulability and angiitis; ACA can exert a suppressive impact not only on the anticoagulation protein C and antithrombin III, but also on the vascular endothelial cells to release prostacycline, known as an inducer of hypercoagulability; ACA may allow vascular endothelial cells for the overexpression of adhesion molecules, which, in turn, lead to the adhesion of platelet and inflammatory cells, thereby forming the cellular inflammatory thrombus besides the relative sparing of the vessel wall (24).

The agonistic autoantibodies (agAAB) in reaction with the epitopes restricted to the extracellular loops of the G-protein coupled receptors (GPCR) have been demonstrated to modulate different cardiovascular pathologies (33-36). The binding of agAAB to the receptor results in the induction of downstream signaling cascades (37). The additional GPCR-agonists stimulation of receptor pathways by the agAAB is more likely to cause a prolonged and unphysiological postreceptor effect (38). The pathogenic role of the circulating agAAB has been widely highlighted in animal models and clinical studies (39-42). As for the pathogenesis of TAO, Klein *et al.* (43) showed that the agAAB were detected against the GPCR in 81.8 % of the active TAO patients, with 63.6% displaying the multiple agAAB against the GPCR. Besides, the clustering of the agAAB binding to the extracellular receptor loop1 of the α1-adrenergic and the ETA-receptor was evident in these patients. However, there was no agAAB directed against loop2 of these two receptors. Noteworthy, they administered a five-day treatment with immunoadsorption and observed that the agAAB were effectively removed in 77.8 % of their patients. However, the re-occurrence of the agAAB was reported during the treatment course. It was speculated that the removal of the agAAB can afford to improve vasospasm and microcirculation, as previously characterized by the notable amelioration of pain and digital pulse curves, sharp reduction of transcutaneous oxygen pressure and carbon dioxide pressure, along with ulcer healing (44, 45).

Increased titres of IgG against periodontal pathogens can also account for the pathogenesis of TAO (46). Slavov *et al.* (47) documented that the TAO patients with the persistent immune inflammation showed changes in the synthesis of culture supernatant as well as the serum interleukin (IL)-6, IL-12 and IL-10, and increases in the apoptosis of polymorphonuclear leukocytes. Moreover, they had high concentrations of the circulating immune complexes, including, IgG, IgM, and IgA. An elevation of the plasma endothelin-1 level was linked to the clinical aggravation of TAO symptoms (47). Other biomolecules, namely the P-, E-, and L-selectins, which appear to be responsible for the vascular endothelium increased in TAO. Actually, the treatment of the endothelium with prostaglandin E1 (PGE1) not only can ameliorate the tissue perfusion, but also mitigate the selectin expression that finally prohibits the inflammatory reaction and leukocyte deposition in the vascular wall (48-50). Likewise, in TAO upon the activation of endothelial cells due to lesions, the release of TNF-α may be induced by inflammatory cells, while the expression of intercellular adhesion molecule-1 (ICAM-1), vascular cell adhesion molecule-1 (VCAM-1), and E-selectin increases for the leukocyte adherence (51). Another study documented that the expression of ICAM-1 and VCAM-1 notably elevated in the human umbilical vein endothelial cells (HUVECs) exposed to the sera obtained from the TAO patients as opposed to the healthy smokers and non-smokers. Also, changes in the gene expression of ICAM-1 and VCAM-1 were found to have an association with the duration of smoking as well as daily number of smoked cigarettes, respectively. These findings suggest that endothelia cells are activated during the disease, as characterized by increases in ICAM-1, VCAM-1, and so forth. The potential trigger for this event can be cytokines, especially if generated by activated platelets, free oxygen radicals, and low NO levels (52). 

The aggregates of immunoglobulin and complement, along with the presence of the CD4(+) and CD8(+) T-lymphocytes, CD20(+) B-lymphocytes, and S-100-positive dendritic cells are also indicative of the endothelial activation next to the internal elastic lamina that they all undergo structural alterations (6, 51, 53-56). The development of giant cells and microabscesses is more likely to be accompanied by the mononuclear cell-rich thrombus, as well (57). Besides, lymphocytes (T and B) as well as macrophages were detected in the biopsies of muscle fibers obtained from the TAO patients (6, 56, 58-60). In the study by Kobayashi *et al. *(6) concerning the cell infiltration in the arteries obtained from these patients, a greater extent of the intimal CD4(+) and CD8(+) T lymphocytes, a lesser extent of the CD20(+) B lymphocytes in the vessel walls, and the macrophage infiltration were present in thrombosis and vessel intima. Furthermore, there were the CD68(+) macrophage as well as S-100(+) dendritic cell infiltration in the intimal layer of the arterial walls, and a larger number of the CD3(+) T cells than the CD20(+) B cells. The findings of immunohistochemistry in a TAO patient with impaired multiple large vessels demonstrated the presence of the CD3(+) T cells around the recanalization sites in the abdominal aorta. The CD4(+) T cells were found to the same extent as the CD8(+) T cells (61). Using the scanning electron microscope, it was exhibited that the immune complexes and macrophage were accumulated, and the neutrophil infiltration occurred in the occluded blood vessels of the TAO patients (62). It appeared that, irrespective of the current smoking status among the TAO patients, a foundational increase was observed in the proinflammatory (IL-1β, TNF-α and IL-6), T helper (Th) 1 (IFN-γ and IL-12), Th2 (IL-4, IL-5 and IL-13), and Th17 (IL-17 and IL-23) cytokines in comparison with the non-, ex- and active smokers, which is indicative of the Th1 and Th2 cells involvement in the development of TAO. The presence of the Th17 cytokines denotes the TAO susceptibility in smokers (63). Substantial evidence shows that the T cells in TAO are associated with many functional abnormalities. The T-cell compartment is disturbed and the T-cell subsets are changed by ANA. Moreover, the CD4(+) and CD8(+) T-lymphocytes aggregate alongside the lamina elastica interna and change its structure. In the intimal layer of the arterial walls, the CD3(+) T cells dominate against the CD20(+) B cells. They also accumulate around the recanalization sites in the abdominal aorta. However, the number of the CD4(+) T cells is in balance with that of the CD8(+) T cells. The levels of certain cytokines are increased along with proinflammatory factors, suggesting the actions of Th1, Th2, and Th17 cells are affected. To develop therapeutic approaches regulating the immunological function of T cells, further studies on the basic immunological properties of T cells are required in the pathogenesis of TAO.

**IL-33 release**

IL-33 belongs to the IL-1 cytokine family, being constitutively detected in the nucleus of endothelial and epithelial cells (64), and acting as a ligand for the IL-1 receptor (IL-1R) family member ST2 (65). It was revealed that IL-33 functions throughout the early events of the inflammation, with a considerable action in the host reactions to a variety of pathogens and allergens (65). It has been reported that the TAO patients, especially the active smokers, were found with a significantly higher plasma level of IL-33 versus their counterparts in the control groups (66), denoting the participation of IL-33 in the pathogenesis of TAO. Nevertheless, the mechanism underlying the contribution of smoking to the IL-33 level and TAO still requires more research. How the IL-33 levels play a role in TAO may arise from the endothelial dysfunction. Endothelial cells were determined as a paramount functional target for IL-33 in regulating the allergic inflammation (67). There have been some reports supporting impairments to the endothelial functions during TAO (68-70). More recently, Stojkovic *et al.* (71) showed that IL-33 was able to increase the thrombotic capacity of HUVECs through the ST2- and NFκB-dependent mRNA and protein expression of tissue factor (TF). Impaired endothelial cells produce IL-33 that culminates in the induction of the immune system and accumulation of Th2 cells, thus elevating the local levels of IL-4, IL-5, and IL-13 (64, 65, 72). Moreover, IL-33 could augment the endothelial cell surface ICAM-1 expression, enhance the leukocyte adhesion, and disrupted the endothelial barrier along with the IL-4-induced apoptosis of endothelial cells in a reversible fashion, leading to an uncontrolled progression of the destabilized endothelial barrier and most likely to the aggravation of the inflammatory damage in the long run (73). On the contrary, the IL-33 signaling can afford to protect the cardiovascular system. As an example, IL-33 was demonstrated to improve the formation of atherosclerotic plaques in mice via repressing the conversion of macrophages into foam cells (74) and yet enhancing a shift from the Th1- to Th2-type immune reaction (75). More to the point, it was recorded that IL-33 was of high capability to stabilize the atherosclerotic plaques through the ERK1/2, JNK (JNK1/2 and c-Jun), and PI3K signaling pathways, except for p38 MAPK (76). IL-33 seems to be the most important cytokine for the Th2-mediated host defense and implicated in controlling immune responses to the early events of the inflammation and endothelial dysfunction. Alteration of the IL-33/ST2 pathway can be a potentially new therapeutic target for treating or preventing different inflammatory events in TAO. Nevertheless, the biology of IL-33, including its nuclear impacts, and processing as well as release of IL-33 from cells, is still unknown. Moreover, considering the high number of cellular responses affected by IL-33 and ST2, especially the role of IL-33 cardio-protection, this strategy should be implemented with caution.

**MyD88-dependent TLR signaling pathway**

The IL-33 receptor complex is composed of ST2 and the IL-1R accessory protein (IL-1RAcP) (77). IL-33 is defined as a peculiar ligand for ST2L, a transmembrane protein, the structure of which contains a triad of the extracellular immunoglobulin domains as well as an intracellular Toll/IL-1 receptor (TLR) domain (78). Upon the binding of IL-33 to ST2, the IL-1-related protein kinase (IRAK), tumor necrosis factor receptor associated factor 6 (TRAF6), MAPK, and NFκB are evoked by means of MyD88 that consequently triggers a series of biological functions (78, 79). Previously, it was presented that MyD88 single nucleotide polymorphism (SNP) in the TLR signaling pathway was not only connected to the TAO pathogenesis (80), but also responsible for the process of the endothelial cell injury. Pahwa *et al. *(81) highlighted that the high glucose levels caused an increase in the ROS generation, and accordingly in the TLR2 as well as TLR4 mRNA and protein expression, which, further, stimulated the MyD88-dependent and MyD88-independent TLR signaling pathways, promoting the monocyte adhesion and local inflammatory reaction in the aortic endothelial cells, and most likely ending up in injury. Echavarria *et al. *(82) presented that angiopoietin-1 had a lowering effect on the inflammatory response in the LPS-treated HUVECs through the induction of miR-146b-5p expression, which acts as an inhibitory regulator of the MyD88-IRAK1-TRAF6 signaling pathway. Aplin *et al.* (83) observed that in a mouse model of the aortic vascular injury accompanied with angiogenesis, the overexpression of TLRs, the stimulation of TLR signaling pathways, and the promotion of downstream gene sequences occurred, while differently in the MyD88 knockout mouse cell culture, the induction of angiogenesis was inhibited following aortic injury, which is indicative of the implication of MyD88 in the TAO pathogenesis. Overactivation of TLRs, particularly TLR2 and TLR4, can finally induce the disturbance of immune homeostasis, and therefore elevate the risk for inflammatory disorders and autoimmune diseases, including TAO. Given the evidences implying the role of TLRs in the development and progression of TAO, those antagonists/inhibitors targeting the TLR signaling pathways represent novel therapeutic agents to treat these diseases. Furthermore, the involvement of MyD88 single nucleotide polymorphism warrants further investigations.

**Sympathetic ganglia inflammation**

The other mechanism involved in the development of TAO regards the presence of inflammation in the sympathetic ganglia. In the study by Farzadnia *et al.* (84) on the 19 Caucasian male patients with TAO, the infiltration of lymphocytes, T cytotoxic (T CD8(+)) lymphocytes in particular, was observed in the sympathetic ganglia. Moreover, the neutrophil infiltration occurred in some patients having the below-knee amputation following sympathectomy. However, the sympathetic ganglia were not close to the site of the ischemia as well as vascular damages. This event can be related to the microvasculature surrounding the sympathetic ganglia whereby the inflammatory cells reach the ganglia, which is exactly similar to the neural inflammation within the peripheral neurovascular bundle. Additionally, it appears subsequent to the long-term irritation of the peripheral nerves throughout the ischemia or peripheral vascular damages. Since, it has been recently reported that the chronic irritation of the peripheral nerves can enhance the T CD4(+) lymphocyte infiltration in the sympathetic ganglia of rats (85). The T cytotoxic lymphocytes were prevailing in the sympathetic ganglia of the TAO patients, though. What is more, a great extent of the infiltrated T cytotoxic lymphocytes along with neutrophils in the sympathetic ganglia denotes a possible intracellular infection by pathogens that not only contributes to the development of TAO, but also recruits the pathogen-specific T cells in the sympathetic ganglia of TAO. The existence of the Th lymphocytes in some samples as shown by Farzadnia *et al.* (84) partly supports the role of pathogens, because the Ths (CD4(+)) accumulate owing to inflammation and likely neuronal impairment to reverse the neuronal function (86, 87). The other cause behind sympathetic ganglia inflammation may be *Rickettsia* infection due to a flea or louse bite. The TAO patients frequently present with a circadian rhythmic burning pain that occurs prior to the onset of ischemia in general (11). *Rickettsia* can afford to induce the kallikrein-kinin system (KKS), known as a regulator of pain sensation (tingling and burning) (88). Current evidence has been indicative of the activation of kinin system in TAO (89). *Rickettsia* was exhibited to increase the kinin system receptors through the expression of inflammatory cytokines (90). For example, it was able to trigger the expression of the chemokine (C-X-C motif) ligand 1 (CXCL1) from monocytes (91). Of note, CXCL1 showed a circadian rhythmic expression, potentially irritating the terminals of sympathetic nerves in a direct fashion (53). More importantly, *Rickettsia* causes infections in the microcirculation of the adrenal gland products, which are more likely to account for an imbalance between the sympathetic and parasympathetic systems (92). Accumulating evidence draws attention to the inflammatory changes in the sympathetic ganglia in TAO. Lymphocytes, especially T cytotoxic (T CD8(+)) lymphocytes, and neutrophils were infiltrated in the sympathetic ganglia. The Ths (CD4(+)) also aggregate in the nervous system. However, these events may indicate the presence of an intracellular infection, *Rickettsia *for instance, or the induction of the KKS. Studies suggest the KKS as a mediator of inflammatory responses, thus, it would be reasonable to investigate the link between the KKS and oxidative stress in the pathogenesis of TAO.

**Cyclooxygenase inflammatory pathway**

Vasculitis is defined as a pathological process associated with the inflammatory damage to the blood vessels and likely arises from genetic abnormalities, autoimmune or physical damage. Throughout acute-phase lesions along with occlusive cellular thrombosis, the acute inflammation as an immune system’s response affects all layers of the vessel wall and accordingly TAO can be viewed as a vasculitis (93). In the sodium laurate-intoxicated rats, the endothelial cell function is impaired and the accumulation of platelets occurs in the peripheral vascular beds (94). This model of vasculitis is similar to the evidence manifest in the patients with TAO, gangrene, imputation, the narrowing or complete occlusion of the vessel lumen, and the recanalization of the artery (95, 96). The PGs are involved in the trigger of the inflammatory reaction and their biosynthesis is on the notable rise in the affected tissues. Cyclooxygenase (COX) is the rate-limiting enzyme that controls the first two steps related to the production of PGs. This enzyme has two isoforms, namely COX1 and COX2 (97, 98). Some studies have reported that the ICAM-1 level increases in the TAO patients (51, 69) and the administration of COX2 inhibitors can afford to improve the disease condition (99, 100). Likewise, in the study by Xu *et al.* (101) on modelling the mechanisms of TAO, the plasma urocortin (a locally expressed pro-inflammatory peptide from the corticotrophin-releasing factor (CRF) family), PGE2, and soluble ICAM-1 levels augmented. The expression of urocortin, CRF1 and CRF1α-receptors, COX2, and ICAM-1 was elevated considerably in the rat femoral arteries, as well. The 12-day treatment with exogenous urocortin aggravated the hypercoagulable state and caused an increase in the expression of CRF1α-receptors, COX2, and ICAM-1. More importantly, these changes were reversed by a CRF1-receptor antagonist (NBI-27914) or a non-selective CRF-receptor antagonist (astressin), but not by the CRF2-receptor antagonist (antisauvagine-30) coadministered with exogenous urocortin. Furthermore, the elevation of COX2 and ICAM-1 may account for the aggravation caused by urocortin. Another *in vitro* study by Zhang *et al.* (102) indicated that exogenous urocortin augmented the expression of COX2 and ICAM-1 in the rat aortic endothelial cells exposed to lipopolysaccharide in a time- and concentration-dependent fashion. Similarly, they found that exogenous urocortin exerted an increasing influence on the PGE2 and soluble ICAM-1 levels. These changes were recovered by the CRF2-receptor antagonist, but not by the CRF1-receptor antagonist. Additionally, exogenous urocortin stimulated p38MAPK, and elevated the NF-ĸB nuclear translocation as well as phosphorylation, while on the contrary the ERK1/2, JNK, and Akt pathways remained unchanged in this process. More to the point, *Rickettsia* was shown to activate the COX inflammatory pathway and augment the production of PGE2 that consequently influence vascular permeability and edema (103). Given that PGE2 can induce a hyperalgesic effect in the peripheral nervous system, and a blood clot or an artery spasm in the cardiovascular system (104), PGI2 as an antagonist of PGE2 can alleviate the symptoms of TAO (Figure 2) (11). Since the products of COX2 in particular are likely to resolve inflammation in certain medical conditions, it is worth investigating which products of COX2 can promote this role in TAO. Moreover, throughout the course of an inflammatory response, both the level and the profile of PG synthesis show significant changes. In TAO setting, these changes are in need for more research.

**Impaired development of collateral arteries by interferon **γ** and vascular endothelial growth factor receptor 1 **

The peripheral blood mononuclear cells (PBMCs) has been an emerging treatment approach for TAO due to their role in neovascularization (105), including angiogenesis, vasculogenesis, and arteriogenesis (collateral artery growth). Angiogenesis by the vascular endothelial cell growth factor-A (VEGF-A) as well as vasculogenesis by stem cells are adopted as the two main mechanisms to biologically manage the development of TAO (106, 107). Despite the positive contribution of these treatments to limb saving, they fail to ameliorate claudication (108, 109). This may be correlated to arteriogenesis (development of collateral arteries) which is not influenced during the therapy. Fazeli *et al.* (53) performed an *in vitro *study on PBMCs isolated from the three groups of participants, including the TAO patients, healthy smokers, and healthy non-smokers. They examined the expression of many genes involved in angiogenesis by PBMCs. It was reported that the expression of the CXCL1 gene (an inducer of collateral development) significantly augmented in the TAO patients versus the healthy controls however no marked differences were determined between the smokers and non-smokers. Additionally, the IL-8 (an inducer of collateral development) expression showed a significant rise in the lymphocytes of the TAO patients and smokers as opposed to the non-smokers. On the other hand, no statistically significant disparity occurred in the IFN-γ (an angiogenesis inhibitor) expression between the TAO patients and smokers although the TAO patients as well as smokers presented considerably higher values than the non-smokers did. Moreover, the VEGFR1 (an angiogenesis inhibitor) expression in the TAO patients and smokers displayed a significant elevation versus the non-smokers. There was no substantial difference in the expression of VEGF-A and inducible nitric oxide synthase (iNOS) genes (angiogenesis inducers) among these groups. As documented, CXCL1 besides IL-8 activate the production of progenitor cells from the bone marrow (110, 111). It can be assumed that the increased levels of IFN-γ and VEGFR1 appear as a predicament that prohibits the effective homing of the circulating progenitors to the site of injury in TAO. Of note, in the TAO patient, the number of the circulating progenitor cells is negatively impacted by oxidative stress and subsequent apoptosis (9, 112). Park *et al.* (113) highlighted that the balance between the eNOS-induced NO and ROS plays a pivotal role in the progenitor cell dysfunction through affecting their migration and tube formation. However, they only found decreases in the number of these cells. Fundamental evidence support defects in a variety of progenitor cell populations in TAO. These deficits can exert effects on several aspects of progenitor cell biology, including renewal, differentiation, homing, cytokine production, and neovascularization, by mechanisms that still remain unknown. Understanding how TAO changes progenitor cell signaling and homeostasis may be helpful for the success of cell-based therapies.

**High mobility group box 1 (HMGB 1): a late inflammatory cytokine**

HMGB-1, as a damage-associated molecular pattern protein, is substantially involved in inflammation via triggering and increasing the production and secretion of proinflammatory cytokines, changing the signal transduction and apoptosis/autophagy responses, and expressing the adhesion molecules and matrix-degrading enzymes (114). These enzymes from the matrix metalloproteinase (MMP) family participate in tissue remodelling in various diseases (115). They are mostly produced as the inactive zymogens except for the proteinase stromelysin-3 (MMP-11)**,** which is an active enzyme (116). It has been reported that heavy smoking decreases the expression of MMP-2 and MMP-9 (117). Nevertheless, the circulating levels of MMP-9 appear to be on the rise in TAO (118). In an animal model of TAO, the HMGB-1 blockade was found with promising outcomes in diminishing the pathogenesis of TAO (119). More recently, De Caridi *et al.* (120) demonstrated that the circulating levels of HMGB-1 notably increased in the TAO patients as opposed to the controls and were strongly related to the elevated levels of the soluble ICAM-1. Only MMP-9 was considerably different in the TAO patients versus the healthy non-smokers. The other two enzymes, MMP-2 and MMP-11, were comparable between the TAO and healthy cases. They pinpointed the NF-ĸB activity as an influential factor related to this difference. Indeed, the induction of MMP-9 is independent from the classical macrophage activation and NF-ĸB activity, thus it remains unchanged in the atherosclerotic lesions (121). On the contrary, the MMP-9 promoter carries the binding sites of activator protein 1 (AP-1) and NF-ĸB, which are implicated in the synergistic trigger of MMP-9 by growth factors as well as inflammatory cytokines in vascular smooth muscle cells (122). In another study, a net increase occurred in the IL-6 serum levels of the TAO patients (47). Also, HMGB-1 can afford to activate IL-6, thus an immune inflammation may persist in TAO (123). It would be appropriate for the future studies to explore a potential link between HMGB-1 and oxidative stress in the pathogenesis of inflammation and angiogenesis associated with TAO. Manipulating the HMGB-1 activity may afford a novel strategy for treating TAO.

## Conclusion

Evidence shows that the immune system is implicated in developing or progressing TAO via the oxidative stress, T cell-related immune injury, IL-33 release, MyD88-dependent TLR signaling pathway, sympathetic ganglia inflammation, COX inflammatory pathway, impaired development of collateral arteries by IFN-γ and VEGFR1, and HMGB 1. The recognition of the molecular pathways contributing to the development of TAO may lead to the novel molecular therapies that can restore the immunologic homeostasis impaired during the disease. Also, the biological agents targeting these mechanisms can be used to screen out the vulnerable population, of note the smokers, at risk without established TAO to improve their medical condition. This immunoreactive point of view offers a bold vision for the management of TAO in the future. Intriguingly, it would be helpful for the better management of TAO to investigate how to convert the immune environment to an angiogenic response by either eliminating proinflammatory cytokines or using angiogenic molecules.
